# N-terminal acetylation can stabilize proteins independent of their ubiquitination

**DOI:** 10.1038/s41598-023-32380-3

**Published:** 2023-04-01

**Authors:** Bert van de Kooij, Evert de Vries, Rogier W. Rooswinkel, George M. C. Janssen, Frédérique K. Kok, Peter A. van Veelen, Jannie Borst

**Affiliations:** 1 Department of Human Genetics, Leiden University Medical Center, Leiden, the Netherlands; 2 Division of Tumor Biology and Immunology, The Netherlands Cancer Institute, Amsterdam, the Netherlands; 3 Department of Immunology and Oncode Institute, Leiden University Medical Center, Leiden, the Netherlands; 4 Center for Proteomics and Metabolomics, Leiden University Medical Center, Leiden, the Netherlands; 5 Leiden Academic Centre for Drug Research, Leiden, the Netherlands; 6 Present Address: Department of Medical Oncology, University Medical Center Groningen, Groningen, the Netherlands

**Keywords:** Biochemistry, Molecular biology

## Abstract

The majority of proteins in mammalian cells are modified by covalent attachment of an acetyl-group to the N-terminus (Nt-acetylation). Paradoxically, Nt-acetylation has been suggested to inhibit as well as to promote substrate degradation. Contrasting these findings, proteome-wide stability measurements failed to detect any correlation between Nt-acetylation status and protein stability. Accordingly, by analysis of protein stability datasets, we found that predicted Nt-acetylation positively correlates with protein stability in case of GFP, but this correlation does not hold for the entire proteome. To further resolve this conundrum, we systematically changed the Nt-acetylation and ubiquitination status of model substrates and assessed their stability. For wild-type Bcl-B, which is heavily modified by proteasome-targeting lysine ubiquitination, Nt-acetylation did not correlate with protein stability. For a lysine-less Bcl-B mutant, however, Nt-acetylation correlated with increased protein stability, likely due to prohibition of ubiquitin conjugation to the acetylated N-terminus. In case of GFP, Nt-acetylation correlated with increased protein stability, as predicted, but our data suggest that Nt-acetylation does not affect GFP ubiquitination. Similarly, in case of the naturally lysine-less protein p16, Nt-acetylation correlated with protein stability, regardless of ubiquitination on its N-terminus or on an introduced lysine residue. A direct effect of Nt-acetylation on p16 stability was supported by studies in NatB-deficient cells. Together, our studies argue that Nt-acetylation can stabilize proteins in human cells in a substrate-specific manner, by competition with N-terminal ubiquitination, but also by other mechanisms that are independent of protein ubiquitination status.

## Introduction

The behaviour of a cell is largely determined by the composition of its proteome, which is shaped by the rate of protein production together with protein lifetime in the cell. Protein lifetime is determined by a combination of parameters that affect susceptibility to degradation by the proteasome or other proteolytic machineries^1^, and varies widely between proteins^2^. Ubiquitination is a major parameter of protein stability. Ubiquitin is a 76 amino acid protein that is transferred to its substrates by a three-step cascade that sequentially involves an E1 ubiquitin activating enzyme, an E2 ubiquitin conjugase, and an E3 ubiquitin ligase^3^. Ubiquitin is covalently linked to a free amino-group, most frequently on the side chain of a lysine residue, but in some cases on the N-terminal α-amino-group^4^. Ubiquitin itself can be ubiquitinated on each of its seven lysine (K) residues and its N-terminus, which generates ubiquitin chains with varying length and topology. Poly-ubiquitin chains linked via K48 function as a strong degradation signal that is directly recognized by the 26S proteasome.

N-terminal acetylation (Nt-acetylation) is a second parameter associated with protein stability^5^. Nt-acetylation is the covalent transfer of an acetyl-group from Acetyl-Coenzyme A to the α–amino-group of the N-terminal residue^6^. It is conserved in all eukaryotic cells and is an irreversible modification that is mediated by N-terminal acetyltransferase (NAT) complexes. Human cells express seven NAT complexes (NatA-NatF and NatH), five of which are associated with the ribosome and predominantly acetylate their substrates co-translationally. NatD specifically targets histones, but each of the other ribosomal NATs acetylate a specific set of substrates that is selected on basis of the first two amino acids of the nascent protein. NatA is selective for proteins from which the initiator methionine has been removed by methionine aminopeptidases (MetAPs), which occurs when the residue on position 2 is an A, C, G, P, S, T or V^7,8^. NatB targets proteins with a retained initiator methionine that is followed by a D, E, N or Q residue. Together, NatA and NatB target the majority of Nt-acetylation substrates, while the remaining co-translational Nt-acetylation is performed by NatC and NatE that acetylate a relatively broad and somewhat overlapping substrate repertoire. Proteomic studies indicate that collectively, NATs target about 80–90% of all human proteins, making Nt-acetylation one of the most prevalent protein modifications in the cell^9^. Not surprisingly therefore, a germline mutation in the gene encoding the NatA catalytic subunit was found to have catastrophic consequences and be responsible for Ogden syndrome, a severely pathogenic disorder resulting in death prior to the age of two years^10^.

Early biochemical studies indicated that Nt-acetylation can enhance protein stability. This conclusion was based on the observation that Nt-acetyl-free proteins were more susceptible to ubiquitin-dependent degradation in rabbit reticulocyte lysates than Nt-acetylated proteins^11–13^. Consistently, a more recent study in human cell-lines showed that Nt-acetylated variants of the protein THOC7 had a substantially longer half-life than Nt-acetyl-free variants^14^. Furthermore, global quantitative mass spectrometry (MS) followed by targeted validation experiments in *Arabidopsis thaliana* indicated that in plant cells the majority of NatA substrates were destabilized by depletion of NatA, or of the NatA activating protein HYPK^15,16^. Similarly, loss of Nt-acetylation upon NatA deletion in yeast correlated with a significant reduction in turnover time of NatA substrates^17^. Nt-acetylation blocks ubiquitin conjugation to the N-terminus^18^, which has been suggested as a mechanism by which it can prevent protein degradation^11^. In agreement, mutating the N-terminus of mouse p19^ARF^ protein to promote Nt-acetylation prevented N-terminal ubiquitination, which correlated with enhanced stability^19^.

Nevertheless, a protein-stabilizing function of Nt-acetylation remains debated due to findings that contradict the above-mentioned studies^20^. Whereas THOC7 was stabilized by Nt-acetylation, six other proteins that were analyzed in the same study were equally stable as Nt-acetylated and Nt-acetyl-free variant^14^. Moreover, a high throughput analysis of degradation signals (degrons) in the N-terminal proteome of yeast could not detect a correlation between Nt-acetylation and protein degradation rate, except for specific N-termini starting with the sequence MN or EZ (where Z = E, T, S, D, H, Q, M)^21^. Also, proteomics-based analysis of close to 2000 proteins in human cells did not point to Nt-acetylation as a determinant of protein turnover time^22^. Even more paradoxical, the opposite function of an acetylated N-terminus was identified in yeast, where it can function as a degron that is recognized by the ER-associated E3 ligase Doa10^23,24^. This Nt-acetyl degron function was later confirmed to exist in human cells as well, targeting the protein Rgs2 for ubiquitin-mediated degradation^25^.

Taken together, these contradictory findings suggest that a function of Nt-acetylation in protein stabilization or degradation might be highly dependent on the substrate. We therefore set out to investigate which other substrate-specific parameters determine the impact of Nt-acetylation on protein stability. Here, by methodically changing the Nt-acetylation status of a defined set of model substrates, we show that protein stability is determined in a substrate-specific manner by Nt-acetylation, ubiquitination, or the crosstalk between those modifications.

## Results

### Correlation between Nt-acetylation and protein stability is substrate-dependent

**Figure 1:**
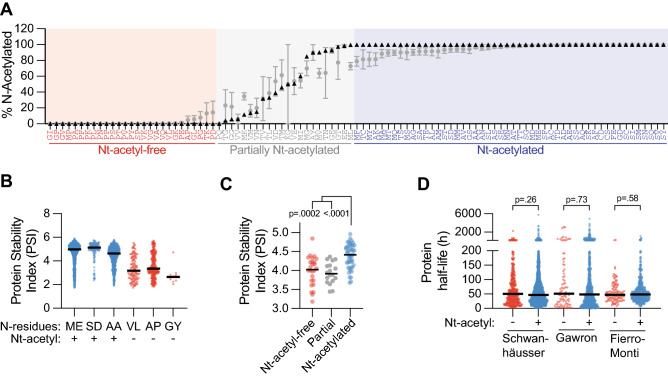
Nt-acetylation correlates with increased stability in case of a modified GFP protein, but this correlation does not apply to all human proteins. (**A**) Three proteome-wide Nt-acetylation studies were mined to extract the average percentage of Nt-acetylation for each detected protein. Subsequently, proteins with identical N-terminal dipeptide sequences were grouped. Plotted are the median (black triangles) and mean ± SEM (grey circles) percentages of Nt-acetylation for each dipeptide sequence group. Only dipeptide sequence groups for which at least two different substrates were detected by the combined Nt-acetylation studies were included. (**B**) Protein stability, as indicated by the Protein Stability Index (PSI), was determined for ~ 50,000 GFP variants that differ with regards to their N-terminal sequence (Timms et al.)^26^. Sequence variants carrying the indicated N-terminal dipeptide sequences were grouped, and the PSI score for each variant was plotted. Black line indicates the median. (**C**) All GFP variants from Timms et al. were grouped according to the N-terminal dipeptide sequence, divided into Nt-acetylation susceptibility bins, and the average PSI score for each group was plotted. Black line indicates the median (Kruskal–Wallis ANOVA, post-hoc Dunn’s). (**D**) The proteins detected in the proteomic studies of Schwanhäusser et al.^2^, Gawron et al.^22^, and Fierro-Monti et al.^30^ were divided into Nt-acetyl-free or Nt-acetylated groups, based on their N-terminal dipeptide sequence and associated Nt-acetylation susceptibility as described in panel A. For each protein the half-life determined by the indicated studies was plotted. Black line indicates the median (Kolmogornov–Smirnov test).

To study the correlation between Nt-acetylation and protein stability, we analyzed a dataset recently published by Timms et al.^26^. The authors fused the 24 N-terminal amino acids of the major isoform of every human protein to GFP, and used a high-throughput approach to determine the stability of each of the ~ 50,000 resulting GFP variants. Hereby, differences in GFP protein stability can be directly attributed to the specific N-terminal sequence. We first aimed to assign Nt-acetylation susceptibility to each of the N-terminal sequences, and therefore compiled the data of three independent MS studies that determined the Nt-acetylation status of the human proteome^27–29^. We grouped all proteins based on N-terminal dipeptide sequence, referring to the mature N-terminus, so without the initiator methionine in case this is followed by an A, C, G, S, T, V or P residue, but including the initiator methionine in all other cases^7,8^. Dipeptide sequence groups for which no proteins or only one protein was detected were excluded. We calculated the median percentage of Nt-acetylation of the resulting 99 dipeptide sequences (Fig. 1A). N-terminal dipeptide sequences were divided into three groups: Nt-acetyl-free (0% median Nt-acetylation; 27 sequences), partially Nt-acetylated (0.125–99.75% median acetylation; 22 sequences) and completely Nt-acetylated (100% median acetylation; 50 sequences; Fig. 1A).

Subsequently, we plotted the Protein Stability Index (PSI) of selected N-terminal sequence GFP variants from the Timms et al*.* data according to their predicted acetylation status: completely Nt-acetylated (ME, SD and AA) or Nt-acetyl-free (VL and AP) (Fig. 1B). The PSI score, as determined by Timms et al., is a measure for protein stability derived from the abundance of a given GFP variant in six stability bins, and ranges between 1 (maximally unstable) and 6 (maximally stable)^26^. The median PSI score for the Nt-acetylation-prone GFP variants was significantly higher than for the Nt-acetylation-resistant GFP variants (Fig. 1B). The PSI score for [GY]GFP variants was included, since this sequence reportedly acts as a strongly destabilizing N-terminal degron^26^. The Nt-acetylome data did not predict Nt-acetylation susceptibility of the GY sequence, but it was categorized as a poor Nt-acetylation substrate given the low level of Nt-acetylation of most proteins with an N-terminal glycine (Fig. 1A). Consistently, [GY]GFP grouped with unacetylated GFP variants as less stable than the acetylated variants (Fig. 1B).

To generalize the findings from these six dipeptide sequences to all 99 N-terminal dipeptide sequence groups, we averaged the PSI score of all GFP variants starting with the same N-terminal dipeptide sequence, and binned these averages in the three Nt-acetylation susceptibility groups. GFP variants that are optimal Nt-acetylation substrates had a significantly higher PSI score than GFP variants that are less optimal or poor Nt-acetylation substrates (Fig. 1C). Taken together, we conclude that for GFP as a model substrate, Nt-acetylation correlates with increased protein stability.

To extend our findings beyond the GFP model substrate, we analyzed three mass spectrometry studies that measured the individual stability of most proteins in the human proteome^2,22,30^. We divided the proteins into Nt-acetyl-free and Nt-acetylated groups based on their N-terminal dipeptide sequence and plotted protein half-life as determined in each study. The median protein half-life was similar for the Nt-acetylated and Nt-acetyl-free substrate groups, in all three studies (Fig. 1D). Thus, unlike the focused study on modified GFP, proteome-wide stability measurements did not reveal a correlation between protein stability and Nt-acetylation status, suggesting that Nt-acetylation is not a general determinant of protein stability applying to all human proteins.

### Nt-acetylation, but not ubiquitination, correlates with HA-GFP stability

**Figure 2:**
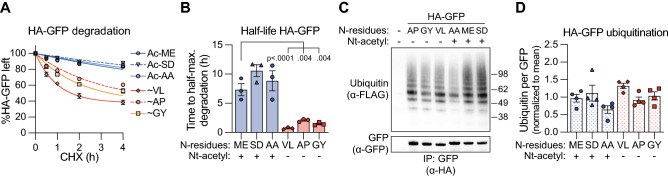
Nt-acetylation correlates with increased stability of HA-GFP, while it does not detectably affect its ubiquitination. (**A**) HEK 293T cells were transfected to express BFP as a reference protein, together with HA-GFP variants carrying indicated N-terminal dipeptide sequences (”Ac-” = Nt-acetylated, “ ~ ” = Nt-acetyl-free). Cells were cultured in presence of CHX (50 μg/ml) for the indicated time periods, followed by flow cytometric analysis. GFP fluorescence intensity was normalized to BFP fluorescence intensity, and set to 100% at the 0 h time point. Data points were connected by a one-phase decay curve fit (n = 3; mean ± SEM). (**B**) Based on the data in panel A, the half-life of the indicated HA-GFP variants was determined (n = 3; mean ± SEM; ratio paired t-test, two-tailed). (**C**, **D**) HEK 293T cells were transfected to express indicated HA-GFP variants and FLAG-tagged ubiquitin, followed by lysis under denaturing conditions. Next, HA-GFP was immunoprecipitated and the precipitates were analyzed by immunoblotting with antibodies to the FLAG tag and GFP. Panel C shows a representative image, panel D shows the quantification of the ubiquitin signal, normalized to the GFP signal (n = 4; mean ± SEM).

We next aimed to understand which substrates might be susceptible to Nt-acetylation-mediated stabilization, and by which mechanism such stabilization occurs. We started with GFP as a model substrate to validate the relationship between Nt-acetylation and stability described above. Wild-type GFP is a highly structured protein that has a very long half-life^31–33^. The GFP variants studied by Timms et al. contained a 24 amino acid extension at the N-terminus, which likely promoted their degradation by acting as an unfolded region that facilitates proteasomal access^34^. To mimic these GFP variants, we added an unfolded region in the form of a double HA tag to the N-terminus of GFP. We tested six HA-GFP variants, carrying either very good (ME, SD or AA), or very poor Nt-acetylation substrate sequences (VL, AP and GY). They were expressed in HEK 293T cells, together with a BFP reference protein, and protein synthesis was inhibited by treatment with cycloheximide (CHX) to assess stability. GFP protein abundance was monitored at different time points after CHX addition by flow cytometry. Nt-acetyl resistant HA-GFP variants were rapidly degraded, while Nt-acetyl prone HA-GFP variants were much more stable in the 4 h CHX chase period (Figs. 2A, S1A). Based on these degradation kinetics, protein half-lives could be determined, which indicated a significantly shorter half-life for the Nt-acetyl resistant variants than for the Nt-acetyl prone HA-GFP variants (Fig. 2B). These experiments thus validate the findings based on analysis of the Timms et al. dataset, and demonstrate that Nt-acetylation correlates with enhanced stability of GFP.

Nt-acetylation has been suggested to determine protein stability by blocking the conjugation of ubiquitin to the N-terminal amino-group^11,19^. To study ubiquitination of the HA-GFP variants, they were expressed in HEK 293T cells together with FLAG-tagged ubiquitin, followed by treatment with proteasome inhibitor to prevent degradation of ubiquitinated protein species. Next, cells were lysed under denaturing conditions to disrupt all non-covalent protein interactions, GFP was immunoprecipitated, and the presence of ubiquitinated GFP species was examined by immunoblotting. We could detect poly-ubiquitinated HA-GFP for all HA-GFP variants, indicating that ubiquitin can be conjugated to one or multiple of GFP’s 18 surface exposed lysine residues (Fig. 2C, see Fig. S6 for uncropped Western blot images). Importantly, the level of ubiquitination was similar between all HA-GFP variants (Fig. 2C,D). Hence, even though Nt-acetylation positively correlated with HA-GFP stability, it did not reduce HA-GFP ubiquitination to an extent that could be detected in our assays. These results therefore suggest that the Nt-acetylated HA-GFP variants were stabilized by a ubiquitination-independent mechanism.

### Nt-acetylation prevents N-terminal ubiquitination and correlates with increased stability of a lysineless Bcl-B mutant, but not of wild-type Bcl-B

For HA-GFP, ubiquitination was clearly not a strong degradation signal, given the long half-life of the (ubiquitinated) Nt-acetylated HA-GFP variants (Fig. 2A,B). We next aimed to study how Nt-acetylation affects protein stability if ubiquitination of the substrate efficiently targets it to the proteasome. To this end, we focused on the anti-apoptotic protein Bcl-B, that we had previously shown to have a short half-life due to K48-linked ubiquitination and consequent proteasomal degradation^35^. We generated N-terminally HA-tagged Bcl-B variants with either an Nt-acetyl-prone (SD), or an Nt-acetyl resistant N-terminal dipeptide sequence (GY and AP). The [AP]HA-Bcl-B variant was guaranteed to be Nt-acetyl-free by the (X)PX-rule^36^. To validate the Nt-acetylation predictions for the other variants we expressed them in HEK 293T cells, followed by immunoprecipitation and analysis by MS. This analysis confirmed that the initiator methionine was removed from both HA-Bcl-B variants, generating a mature N-terminus starting with the sequence GY or SD (Table 1; Fig. S1B, C). Importantly, all [SD]HA-Bcl-B molecules detected were Nt-acetylated, while a large proportion of the [GY]HA-Bcl-B molecules were Nt-acetyl-free, in agreement with the Nt-acetylation predictions based on N-terminal sequence (Table 1; Fig. S1B, C).

**Table 1:** MS-verified Nt-acetylation status of Bcl-B and p16 variants.

Protein	Cell-line	Met removed?	Nt-acetyl
[GY]-HA-Bcl-B	WT	Yes	±
[SD]-HA-Bcl-B	WT	Yes	+
Bcl-B-HA	WT	Partial	+
[ME]p16-HA	WT	No	+
[ME]p16-HA	NatBΔ + EV	No	±
[ME]p16-HA	NatBΔ + NatB	No	+
[GY]p16-HA	WT	Yes	±
[SD]p16-HA	WT	Yes	+
Ubi-[ME]p16-HA	WT	X	+
Ubi-[GY]p16-HA	WT	X	–

Table summarizing the Nt-acetylation status of all Bcl-B and p16 variants that were analyzed by MS (also see Supplementary Figs. 1B,C; 2B; 3A–C and 5A,B). The “±” sign indicates that Nt-acetylated as well as Nt-acetyl-free tryptic peptides were detected. Also indicated is whether the initiator methionine was retained or removed. All experiments were done in HEK 293T cells, either wild-type (WT), or deficient for the NAA20 subunit of NatB (NatBΔ). The latter cell-line was reconstituted with an empty vector (+ EV) or NAA20 cDNA (+ NatB).

**Figure 3:**
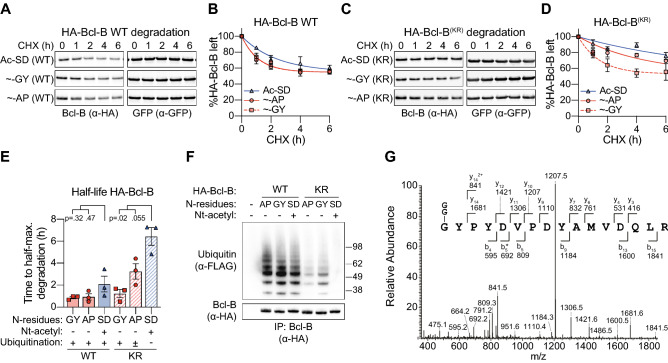
Ubiquitination, rather than Nt-acetylation, determines Bcl-B protein stability. (**A**,** B**) HEK 293T cells were transfected to express GFP as a reference protein, together with HA-Bcl-B variants carrying indicated N-terminal dipeptide sequences (”Ac-” = Nt-acetylated, “ ~ ” = Nt-acetyl-free). Cells were cultured in presence of CHX (50 μg/ml) for the indicated time periods, followed by immunoblot analysis with antibodies to the HA tag and to GFP. HA-Bcl-B signals were quantified and corrected for GFP signal intensity, and the resulting value at the 0 h time point was set to 100%. Data points were connected by a one-phase decay curve fit. Panel A shows a representative Western blot image, panel B shows the quantification (n = 3; mean ± SEM). (**C**, **D**) As in A and B, but for a lysineless (KR) HA-Bcl-B mutant (n = 3; mean ± SEM). (**E**) Based on the data in panels B and D, the half-lives of the indicated HA-Bcl-B variants were determined (n = 3; mean ± SEM, One-way ANOVA, post hoc Dunnett’s). (**F**) HEK 293T cells were transfected to express indicated HA-Bcl-B variants and FLAG-tagged ubiquitin, followed by lysis under denaturing conditions. Next, HA-Bcl-B was immunoprecipitated and the precipitates were analyzed by immunoblotting. A representative image of two independent biological replicates is shown. (**G**) [GY]HA-Bcl-B(KR) was precipitated from HEK 293T lysates, trypsin-digested and analyzed by MS. Shown is the tandem mass spectrum of the N-terminal tryptic peptide, containing a di-glycine (GG) modified N-terminus. 2 +  = doubly charged, * = minus H_2_O.

HA-Bcl-B variants were expressed in HEK 293T cells, together with untagged GFP as a transfection and stable protein control. Protein synthesis was inhibited for different time periods with CHX, and HA-Bcl-B protein levels were monitored by immunoblotting of total cell lysates. HA-Bcl-B was rapidly degraded, reaching a plateau of about 50% of the total protein level present at the start of the CHX chase (Fig. 3A,B). Interestingly, the kinetics of degradation were similar between the Nt-acetylated [SD]HA-Bcl-B and the Nt-acetyl-free [GY] and [AP]HA-Bcl-B (Fig. 3A,B). We therefore conclude that for WT Bcl-B, Nt-acetylation status does not correlate with its stability.

We hypothesized that the strong degradation signals derived from K48-linked ubiquitination of HA-Bcl-B overruled a potential stabilizing function of Nt-acetylation^35^. To assess this, we mutated all lysine residues to arginine (Bcl-B^(KR)^) to prevent lysine ubiquitination of HA-Bcl-B. Next, we performed a CHX chase assay to assess the stability of HA-Bcl-B^(KR)^ variants carrying an [SD], [GY] or [AP] sequence at the N-terminus. Interestingly, degradation of Nt-acetylated [SD]HA-Bcl-B^(KR)^ was substantially slower than of Nt-acetyl-free [GY]HA-Bcl-B^(KR)^, which was degraded as efficiently as WT HA-Bcl-B variants (Fig. 3C–E). Hence, in case of lysineless HA-Bcl-B, Nt-acetylation correlates with increased stability, in contrast to what was found for WT HA-Bcl-B. Notably, the Nt-acetyl-free [AP]HA-Bcl-B^(KR)^ variant was only mildly destabilized compared to [SD]HA-Bcl-B^(KR)^, and more stable than [GY]HA-Bcl-B^(KR)^ (Fig. 3C–E). Thus, N-terminal sequence, in addition to Nt-acetylation status, affects the stability of HA-Bcl-B^(KR)^.

To study the interplay between Bcl-B stability, Nt-acetylation and ubiquitination, the various HA-Bcl-B mutants were assessed for their ubiquitination status. They were expressed in HEK 293T cells together with FLAG-tagged ubiquitin, immunoprecipitated, and analyzed by immunoblotting. WT HA-Bcl-B was ubiquitinated, as found before, and the level of ubiquitination was independent of its Nt-acetylation status (Fig. 3F)^35^. The Nt-acetyl-free [GY] and [AP] HA-Bcl-B^(KR)^ variants were also ubiquitinated, but the Nt-acetylated [SD]HA-Bcl-B^(KR)^ was not (Fig. 3F). Since lysine residues are absent in HA-Bcl-B^(KR)^, ubiquitin was likely conjugated to the α-amino group at the N-terminus, but this was blocked when the N-terminus was Nt-acetylated. To examine N-terminal ubiquitination of Bcl-B, the [GY]HA-Bcl-B^(KR)^ variant was isolated from HEK 293T cells by immunoprecipitation, digested with trypsin, and analyzed by mass spectrometry. A tryptic peptide of the N-terminus modified with a ubiquitin-derived di-glycine remnant was detected, thus providing direct evidence for N-terminal ubiquitination (Fig. 3G). To test whether the native, rather than HA-tagged N-terminus of Bcl-B could also be ubiquitinated, we generated C-terminally HA-tagged Bcl-B variants, either WT or lysineless. All ubiquitination was lost from Bcl-B-HA upon mutation of its lysine residues, suggesting that the native N-terminus of Bcl-B cannot act as a ubiquitin acceptor site (Fig. S2A). Consistently, mass spectrometric analysis of Bcl-B-HA revealed that the N-terminus was completely acetylated, despite it having a suboptimal Nt-acetylation substrate sequence (VD; Table 1, Fig. S2B).

The [AP]HA-Bcl-B^(KR)^ variant carried less ubiquitin than the [GY]HA-Bcl-B^(KR)^ variant, suggesting an impact of N-terminal sequence on N-terminal ubiquitination of these Nt-acetyl-free proteins (Fig. 3F). This also explains the increased stability of the [AP]HA-Bcl-B^(KR)^ variant compared to the [GY]HA-Bcl-B^(KR)^ variant (Fig. 3C–E). Taken together, these data demonstrate that ubiquitination, but not Nt-acetylation, directly correlates with Bcl-B half-life (Fig. 3E), suggesting it is the major determinant of Bcl-B protein stability. The Nt-acetylated Bcl-B variant was stabilized compared to Nt-acetyl-free Bcl-B variants only if lysine ubiquitination was prevented, and our collective data argue that this was caused by Nt-acetylation blocking ubiquitin-conjugation to the N-terminus.

### Nt-acetylation, but not N-terminal or enforced lysine ubiquitination, correlates with stability of the naturally lysineless protein p16

**Figure 4:**
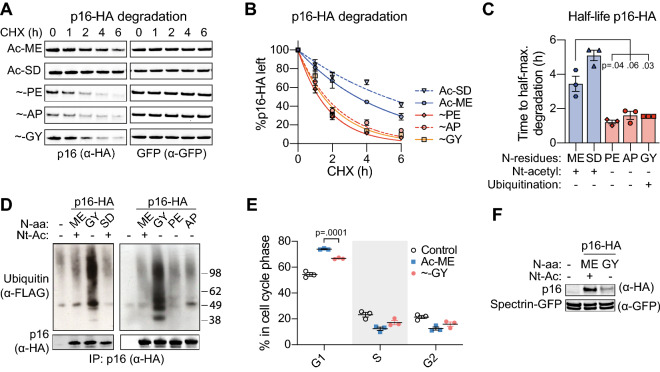
Nt-acetylation correlates with stability of the naturally lysineless protein p16. (**A**, **B**) HEK 293T cells were transfected to express GFP as a reference protein, together with p16-HA variants carrying indicated N-terminal dipeptide sequences (”Ac-” = Nt-acetylated, “ ~ ” = Nt-acetyl-free). Cells were cultured in presence of CHX (50 μg/ml) for the indicated time periods, followed by immunoblot analysis with antibodies to the HA tag and to GFP. P16-HA signals were quantified and corrected for GFP signal intensity, and the resulting value at the 0 h time point was set to 100%. Data points were connected by a one-phase decay curve fit. Panel A shows a representative Western blot image, panel B shows the quantification (n = 3; mean ± SEM). (**C**) Based on the data in panel B, the half-lives of the indicated p16-HA variants were determined (n = 3; mean ± SEM, Ratio paired t-test, two-tailed). (**D**) HEK 293T cells were transfected to express indicated p16-HA variants and FLAG-tagged ubiquitin, followed by lysis under denaturing conditions. Next, p16-HA was immunoprecipitated and the precipitates were analyzed by immunoblotting. A representative image of three independent biological replicates is shown. (**E**) U-2 OS cells were transfected to express indicated p16-HA variants, followed by DNA staining with propidium iodide, and cell cycle analysis by flow cytometry (n = 3; mean ± SEM, Paired t-test, two-tailed). (**F**) Cell lysates from cells described in panel E were analyzed by immunoblotting. Spectrin-GFP is a transfection and loading control.

So far, we found that Nt-acetylation correlated with increased stability of HA-GFP and of a lysineless mutant of Bcl-B. To extend our findings to a natural substrate, rather than an engineered one, we selected the naturally lysineless Cyclin-Dependent Kinase (CDK) inhibitor protein p16-ink4A (*CDKN2A*), referred to as p16. The native N-terminus of p16 contains the Nt-acetyl-prone [ME] dipeptide sequence. P16 was tagged with HA at the C-terminus to facilitate isolation and detection. Nt-acetyl prone [SD]p16-HA, as well as three Nt-acetyl resistant [PE], [AP] and [GY] variants were included in the study. The [PE] and [AP] variants are Nt-acetyl-free according to the (X)PX rule, but for the other variants we assessed their Nt-acetylation status by MS. This analysis demonstrated that the [ME] and [SD] variants were completely Nt-acetylated, whereas the [GY]p16-HA variant was largely Nt-acetyl-free (Fig. S3A–C). The stability of all p16-HA variants was examined by a CHX chase assay. All p16-HA variants were degraded in the 6 h CHX chase period, but the Nt-acetylated [ME] and [SD] variants had a significantly longer half-life than the Nt-acetyl-free [PE], [AP] and [GY] variants (Fig. 4A–C). Hence, p16 protein stability directly correlates with its Nt-acetylation status.

Next, we examined the ubiquitination status of all p16-HA variants. Nt-acetylated [ME] and [SD]p16-HA did not carry any ubiquitin, consistent with the absence of acceptor sites. Nt-acetyl-free [GY]p16-HA was ubiquitinated, but Nt-acetyl-free [PE] and [AP]p16-HA were not (Fig. 4D). Hence, similar to what was observed for Bcl-B, the N-terminal sequence can affect N-terminal ubiquitination independent of Nt-acetylation. The proteasome inhibitor MG132 impeded degradation of the [ME] and [PE]p16-HA variants, indicating that it was proteasomal, even though the proteins were not ubiquitinated (Fig. S3D). Collectively, these data suggest that Nt-acetylation stabilizes the lysine-less p16-HA protein, independent of its competition with N-terminal ubiquitination.

P16 is a frequently mutated tumor suppressor gene that induces a cell-cycle arrest by inhibiting CDKs^37^. To determine if Nt-acetylation status affects p16 function, we expressed [ME]p16-HA or [GY]p16-HA in U-2 OS cells that do not endogenously express p16 and go into G1 cell cycle arrest upon p16 introduction^38^. Cell cycle distribution was determined by flow cytometric analysis of DNA content following propidium iodide staining. A significantly larger fraction of cells was in G1 phase in the [ME] than in the [GY]p16-HA transfected population (Fig. 4E). Although equal amounts of cDNA were transfected for both p16 variants, steady state protein levels of [GY]p16-HA were lower than of [SD]p16-HA, consistent with its reduced half-life (Fig. 4F). Hence, Nt-acetylation can regulate p16 function by virtue of its effect on protein stability.

**Figure 5:**
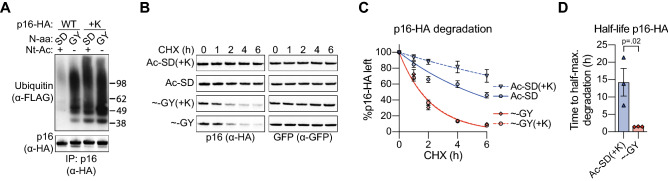
Introducing a lysine ubiquitin acceptor site does not affect p16 stabilization by Nt-acetylation. (**A**) HEK 293T cells were transfected to express indicated p16-HA variants in WT or + K versions and FLAG-tagged ubiquitin, followed by lysis under denaturing conditions. Next, p16-HA was immunoprecipitated and the precipitates were analyzed by immunoblotting. A representative image of three independent biological replicates is shown. (**B**,** C**) HEK 293T cells were transfected to express GFP as a reference protein, together with p16-HA variants carrying indicated N-terminal dipeptide sequences (”Ac-” = Nt-acetylated, “ ~ ” = Nt-acetyl-free). Cells were cultured in presence of CHX (50 μg/ml) for the indicated time periods, followed by immunoblot analysis with antibodies to the HA tag and to GFP. P16-HA signals were quantified and corrected for GFP signal intensity, and the resulting value at the 0 h time point was set to 100%. Data points were connected by a one-phase decay curve fit. Panel B shows a representative Western blot image, panel C shows the quantification. Note that the p16-HA (+ K) variants were studied in side-by side experiments with the p16 variants described in Fig. 4A, B. The Western blot and quantification for the ~ GY variant depicted in Fig. 4A, B are shown here again for comparison purposes (n = 3; mean ± SEM). (**D**) Based on the data in panel C, the half-lives of the indicated p16-HA variants were determined (n = 3; mean ± SEM; Ratio paired t-test, two-tailed).

Finally, we investigated whether having a ubiquitin-acceptor site other than the N-terminus would change the impact of Nt-acetylation on p16 stability. Therefore, a lysine residue was introduced in [SD] and [GY]p16-HA close to the mature N-terminus (+ K). This lysine residue did function as a ubiquitin acceptor site in p16, as evidenced by ubiquitination of the Nt-acetylated [SD]p16-HA (+ K) variant (Fig. 5A). The level of ubiquitination of [SD]p16-HA (+ K) was similar to that of lysineless [GY]p16-HA, indicating that N-terminal ubiquitination and lysine ubiquitination of p16 occurred with similar efficiencies (Fig. 5A). Interestingly, [SD]p16-HA was not destabilized by this lysine ubiquitination and still had a significantly longer half-life than [GY]p16-HA, despite the similar levels of ubiquitination (Fig. 5B–D). Thus, the data taken together indicate that Nt-acetylation regulates p16 protein stability, and the effect of ubiquitination is negligible. This conclusion is based on the findings that (1) an acetyl-free N-terminus acts as a degron in p16 independent of ubiquitination, and (2) forcing p16 ubiquitination by introducing a ubiquitin acceptor site does not overcome the stabilizing effect of Nt-acetylation.

### Nt-acetylation, not N-terminal sequence, is the key determinant of p16 protein stability

Throughout our studies, Nt-acetylation status was altered by changing the N-terminal sequence. To exclude the possibility that these changes directly affected protein stability, we set out to block Nt-acetylation without altering the substrate protein. As a first approach, we fused ubiquitin to the N-terminus of the p16-HA variants, which is efficiently removed by ubiquitin hydrolases during translation^39^. This was hypothesized to shield the N-terminus of p16 long enough to prevent co-translational Nt-acetylation. However, analysis by MS indicated that [ME]p16-HA derived from a ubiquitin-[ME]p16-HA fusion protein was still completely Nt-acetylated (Table 1). Hence, co-translational Nt-acetylation can still occur after ubiquitin removal during protein synthesis, or p16 can be efficiently modified by post-translational Nt-acetylation. Consistent with its Nt-acetylation status, the half-life of [ME]p16-HA derived from a ubiquitin-fusion protein was identical to the half-life of [ME]p16-HA that had not been fused to ubiquitin, and significantly longer than the half-life of Nt-acetyl-free [GY]p16-HA derived from a ubiquitin-fusion protein (Fig. S4A–C).

**Figure 6:**
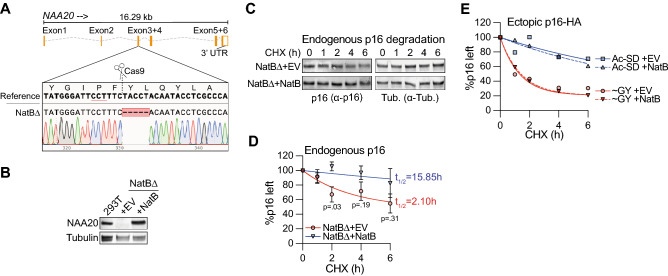
Nt-acetylation, not N-terminal sequence, determines p16 protein stability. (**A**) The region surrounding the Cas9 target site in the *NAA20* gene was PCR-amplified from a clonal NatBΔ HEK 293T cell line and analyzed by sequencing. Shown is the *NAA20* gene structure, inset shows a zoom of the Cas9-target site with the PAM sequence underlined in red. (**B**) Immunoblot analysis of lysates from the HEK 293T parental cell line, and the clonal NatBΔ HEK 293T cell line reconstituted with either an empty vector (+ EV) or NAA20 cDNA (+ NatB). (**C**, **D**) HEK 293T NatBΔ cells, reconstituted with either an empty vector (+ EV) or with NAA20 cDNA (+ NatB), were treated with CHX for indicated time periods, followed by immunoblot analysis of endogenous p16 protein levels. Tubulin was used as a stable protein and loading control. A representative Western blot image is shown in panel C. Panel D shows the quantification, which was done by normalizing the p16 signal to the Tubulin signal, the 0 h time point was set to 100%. Data were fitted by a one-phase decay curve fit, which was used to quantify the half-life (t_1/2_). P-values are derived by statistical testing between the values in the + EV and + NatB samples at each time point (n = 3; mean ± SEM; Unpaired t-test, two-tailed). (**E**) As in panel D, but for ectopically expressed p16-HA variants (n = 3; mean ± SEM).

As a second approach, we aimed to prevent p16 Nt-acetylation by generating a HEK 293T cell-line that was deficient for NatB, the NAT complex that targets the N-terminal ME-sequence for acetylation. CRISPR/Cas9 technology was used to generate a clonal population carrying a 5 bp deletion in all alleles of the *NAA20* gene that encodes the NatB catalytic subunit (Fig. 6A). Subsequently, the clonal NatBΔ cell-line was infected with lentivirus to introduce either an empty vector (+ EV), or NAA20 cDNA (+ NatB), as such generating isogenic NatB-deficient and -proficient cell-lines. Analysis by Western blot validated NAA20 loss and re-expression at the protein level (Fig. 6B). Next, [ME]p16-HA was expressed in the NatB-proficient (+ EV) and NatB-deficient (+ NatB) cell-lines, and its Nt-acetylation status was determined by MS. Whereas [ME]p16-HA was found to be completely Nt-acetylated in the NatB-proficient cells, its Nt-acetylation was detectably reduced in the NatB-deficient cells (Table 1, Fig. S5A, B). However, the reduction in Nt-acetylation was not complete, as ~ 70% of [ME]p16-HA was still Nt-acetylated in the NatB-deficient cells, despite the complete absence of NAA20 protein. Nevertheless, to reveal how this ~ 30% reduction in Nt-acetylation would affect p16 degradation, we assessed the degradation kinetics of endogenous p16 protein, rather than ectopically expressed p16, in a CHX chase experiment. In NatB-proficient cells, endogenous p16 was hardly degraded in the six hour CHX chase period, while it was clearly degraded in the NatB-deficient cells (Fig. 6C). In each of three individual biological repeats, we found p16 to be more rapidly degraded in the NatB-deficient than in the NatB-proficient cell-line (Fig. S5C). Moreover, despite inter-experimental variation between degradation rates, p16 was significantly more depleted in the NatB-deficient cells than in the NatB-proficient cells at the 2 h time point (Figs. 6D, S5C). Consistently, the half-life of p16, as calculated based on curve fitting the averaged data, was substantially reduced in NatB-deficient compared to NatB-proficient cells (Fig. 6D). To validate that this differential degradation rate in NatB-proficient and -deficient cells was unique to NatB-substrates, we assessed degradation of ectopically expressed [SD]p16-HA and [GY]p16-HA, which are respectively Nt-acetylated by NatA or Nt-acetyl-free. Indeed, presence or absence of NatB did not affect the degradation rate of these p16-HA variants (Fig. 6E). Thus, NatB loss only destabilized NatB-targeted p16, suggesting that destabilization was caused by a lack of Nt-acetylation. Taken together, these data are consistent with Nt-acetylation being a strong determinant of p16 stability.

## Discussion

**Figure 7:**
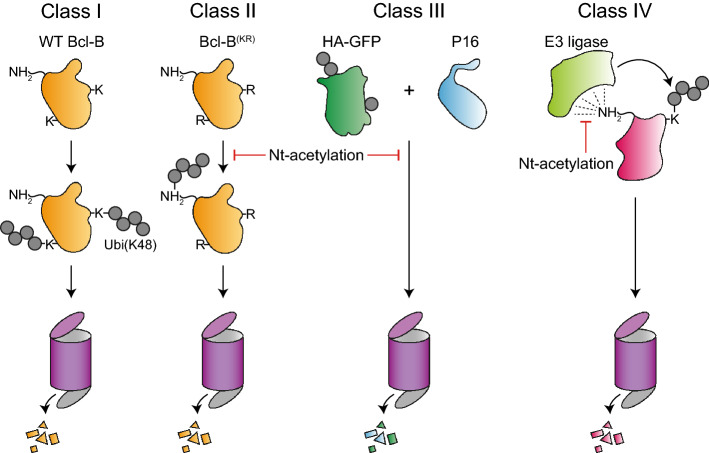
Model describing the interplay between Nt-acetylation, ubiquitination and protein stability. Based on the substrates studied, our data suggest that there are multiple classes of proteins that differ with regards to the impact of Nt-acetylation on protein stability, and its interplay with ubiquitination. Class I proteins, which include wild-type (WT) Bcl-B, are heavily modified by K48-linked ubiquitin chains targeting the substrate for proteasomal degradation. For Class I proteins, ubiquitination is the main determinant of protein stability and there is no impact of Nt-acetylation. Class II proteins are Class I proteins for which lysine ubiquitination is somehow prevented, like for a lysineless Bcl-B mutant (KR). Nt-acetylation inhibits degradation of Class II proteins by preventing ubiquitin conjugation to the N-terminus. Class III proteins might carry ubiquitin, but this does not function as a strong degradation signal. HA-GFP and p16 are Class III proteins. For these substrates, Nt-acetylation enhances protein stability by a mechanism that is independent of ubiquitination. Published literature^21,40,41^ suggests the existence of Class IV proteins, which are stabilized by Nt-acetylation because it prevents recognition by E3 ubiquitin ligases.

Historically, Nt-acetylation has been suggested to inhibit protein degradation^11–13^, but this notion was challenged by more recent studies that either failed to detect a correlation between Nt-acetylation and protein stability, or indicated a protein-destabilizing function of Nt-acetylation^5,21,22^. Here we studied the impact of Nt-acetylation on the stability of a defined set of model substrates, and based on our findings we propose that Nt-acetylation substrates can be divided into different classes (Fig. 7). For class I proteins, stability is determined by ubiquitination on lysine residues and not by Nt-acetylation status. This is exemplified in our study by WT Bcl-B that is constitutively ubiquitinated on lysines and thereby targeted to the proteasome. For class II proteins, stability is also determined by ubiquitination, but due to an absence of (surface-exposed) lysine residues the N-terminus is the ubiquitin acceptor site. Nt-acetylation stabilizes these substrates by blocking N-terminal ubiquitination. For class III proteins, N-terminal acetylation stabilizes the protein independent of its effect on ubiquitination and thereby is the key determinant for stability.

Recent literature suggests the existence of a fourth class of proteins that is stabilized by Nt-acetylation, because it prevents recognition of the N-terminus by specific E3 ubiquitin ligases (Class IV; Fig. 7). For example, a loosely defined N-terminal motif was targeted by the Inhibitor of Apoptosis proteins (IAPs), a family of anti-apoptotic E3 ligases^40^. This motif is predominantly present in NatA substrates, but IAPs only recognize it when it is Nt-acetyl-free^40^. As a second example, genetic inactivation of NatC in human cells resulted in destabilization of NatC substrates, because their acetyl-free N-termini function as degrons that are directly recognized by the ubiquitin ligase UBR4^41^. Finally, proteins carrying the N-terminal dipeptide sequence MN were destabilized in a NatBΔ yeast strain^21^. Nt-acetylation prevented initiator methionine removal and thus the exposure of an N-terminal asparagine residue, which can be converted into a degron directly recognized by the ligase Ubr1^42^. Thus, Nt-acetylation can either directly or indirectly prevent exposure of an N-terminal degron.

Our results demonstrate the existence of different protein classes, but which fraction of the human proteome belongs to each class remains to be determined. This fraction might be small for class II proteins, because N-terminal ubiquitination is relatively infrequent, as indicated by a recent proteomic study that identified 9200 ubiquitinated proteins, of which only 104 carried N-terminal ubiquitin^18^. Moreover, these 104 proteins were generally not stabilized by proteasomal inhibition, suggesting that the N-terminal ubiquitination did not target the substrate for degradation.

Our findings suggest that N-terminal ubiquitination depends on amino acid sequence, in addition to Nt-acetylation status. Lysineless, non-acetylated [AP]HA-Bcl-B^(KR)^, as well as [AP] and [PE]p16-HA, carried less N-terminal ubiquitin than their [GY] counterpart. This may be explained by the recent finding that the GY-sequence can function as a degron^24^. However, the N-terminal GY-sequence did not increase ubiquitination of HA-GFP or WT HA-Bcl-B. More likely therefore, a proline on position 1 or 2 of the mature N-terminus inhibits N-terminal ubiquitination of the [AP]- and [PE] substrates. This is, however, not generalizable to other substrates, because an N-terminal proline was overrepresented in N-terminally ubiquitinated substrates compared to the human proteome^18^. Whether a protein is susceptible to N-terminal ubiquitination is thus dependent on Nt-acetylation status, N-terminal sequence, and additional protein parameters.

Throughout most of our experiments, we mutated the N-terminus to change the Nt-acetylation status of the tested substrates. We aimed to negate confounding effects of the alterations in N-terminal sequence in two ways. First, we used a variety of N-terminal sequences that are either Nt-acetyl-prone (AA, ME, SD) or Nt-acetyl-resistant (VL, GY, AP, PE). Second, we aimed to prevent Nt-acetylation of the NatB substrate p16 by using *NAA20* KO cells. Even though no NAA20 protein was detected in these cells, we found that ~ 70% of p16 was still Nt-acetylated. This is consistent with results from MS-studies in HeLa cells, which showed that the Nt-acetylation status of the majority of NatB-substrates was unchanged after RNAi-mediated depletion of NatB components^29^. Similarly, a substantial fraction of proteins carrying NatC target sequences were still partly or completely Nt-acetylated in NatC KO cell-lines^41^. These results most likely indicate redundancy between the NAT complexes, despite their preference for specific N-terminal sequences.

We show here that Nt-acetylation can stabilize proteins by other mechanisms than blocking N-terminal ubiquitination. These are the class III substrates like HA-GFP and the tumor suppressor protein p16 (Fig. 7). Ubiquitin-independent degradation of p16 was previously reported^43^, and p16 is thus part of an ever-increasing group of proteins that can enter the proteolytic 20S core particle of the proteasome in absence of a ubiquitin signal^44^. Ubiquitin-independent degradation generally requires the presence of a disordered region that can enter the proteasome and pull in the rest of the protein^45^. For the protein Thymidylate Synthase (TS) an acetyl-free, disordered N-terminal region was found to be essential^46^. Fusing the TS N-terminus to GFP resulted in its destabilization. The double HA-tag that we fused to GFP in our studies may have functioned similarly as a disordered region allowing ubiquitin-independent proteasome entry. Hypothetically, reducing structural disorder might be a mechanism by which Nt-acetylation restricts proteasomal entry. It was shown to enhance the helicity of the N-terminus of a-synuclein^47^, and a recent study suggests it promotes the thermostability of cytosolic ribosomal proteins^17^. Alternatively, Nt-acetylation can facilitate protein complex formation or aggregation, either through an effect on protein folding, or directly^48,49^, and as such potentially shield the substrate from the degradation machinery.

In addition to ubiquitination and/or Nt-acetylation, protein half-life is also determined by protein structure, amino acid composition, and hydrophobicity of the N- or C-terminus^2,26,50^. Because the contribution of each parameter to protein stability will vary per substrate, it is not surprising that Nt-acetylation does not emerge as key determinant of protein stability in most proteome-wide studies. Nevertheless, our studies underline that Nt-acetylation should be considered as a protein-stabilizing parameter that contributes to proteome composition, and hence to cellular behaviour.

## Methods

### DNA cloning

All constructs were generated by standard PCR-mediated mutagenesis or restriction-digestion subcloning. Backbones were either pcDNA3 into which an HA-tag sequence was cloned, or pHAN1 or pHAC2, that are variants of pEGFPN1 and pEGFPC2 (Clontech) in which the GFP tag is replaced by a double HA-tag (a kind gift of Lennert Janssen, current address: Department of Cell and Chemical Biology, Leiden University Medical Center, Leiden, the Netherlands). Constructs encoding N-terminally (SD)HA-tagged Bcl-B WT or KR were previously described by van de Kooij et al.^35^. P16 cDNA and spectrin-GFP cDNA were kindly provided by Rob Klompmaker (Division of Cell Biology, The Netherlands Cancer Institute, Amsterdam, The Netherlands) and Indra Shaltiel (current address: Cell Biology and Biophysics Unit, European Molecular Biology Laboratory, Heidelberg, Germany), respectively. The FLAG-ubiquitin construct was previously described by Tait et al.^51^, and was subcloned in a 3 × FLAG-ubiquitin tandem repeat configuration. For ubiquitin-fusion constructs, one copy of ubiquitin cDNA was inserted N-terminally of p16-HA, such that in the protein product the C-terminal glycine of ubiquitin was followed directly by indicated di-peptide sequence of p16-HA. To generate constructs used for making the NatBΔ cell-line, a primer-dimer containing the *NAA20*-targeting sgRNA with sequence 5’- GCGAGGTATTGTAGGTAGAA-3’ was ligated into BsmBI-digested lentiGuide-puro (Addgene #52963)^52^. Furthermore, Cas9 cDNA was PCR amplified from pCW-Cas9 (Addgene #50661)^53^ and ligated into NheI/BamHI digested pcDNA3.1. NatB cDNA was PCR-amplified from reverse-transcribed RNA, and ligated into EcoRI/XhoI digested PMX-IRES-Blasticidin (pMX-Blast).

### Cell culture and transfection

The human osteosarcoma cell line U-2 OS and the human embryonic kidney cell line HEK 293T were obtained from the cryo-storage facility at the Netherlands Cancer Institute, Amsterdam, the Netherlands (original source unknown). Both cell lines were cultured in DMEM, supplemented with 8% FCS and antibiotics at 37 °C, 5% CO_2_. Transfections were performed using polyethyleneimine (PEI) at a 1:3 (w/w) cDNA:PEI ratio. Cells were harvested at 24 h after transfection, or at 48 h after transfection when p16 ubiquitination was assessed. To generate the NatBΔ cell-line, HEK 293T cells were transfected with pcDNA-Cas9 and lentiGuide-puro-NAA20 sgRNA plasmids, followed by selection of transfected cells using puromycin (2 μg/ml, Invivogen). After culturing for more than 7 days to allow for gene editing, single cell clones were plated in 96 well plates, and expanded. A verified NatB KO clone was subsequently retrovirally infected to introduce either a pMX-Blast EV, or a pMX-Blast-NatB. Cells were cultured in the presence of blasticidin (20 μg/ml, Invivogen) to select for transduced cells.

### Immunoprecipitation

To assess protein ubiquitination, cells were treated with 50 μM MG132 (Calbiochem) for 4 h prior to harvest, and subsequently lysed by incubation for 5–10 min in pre-heated denaturing SDS buffer (50 mM Tris–HCl pH 8.0, 1% SDS, 0.5 mM EDTA, 10 mM DTT). SDS was quenched and diluted by adding nine volumes of NP-40 buffer (50 mM Tris‐HCl pH 7.4, 150 mM NaCl, 1% NP‐40, 1 mM PMSF, Roche protease inhibitor cocktail, 1 mM EDTA). Lysates were centrifuged for 15 min at 13.000*g*, 4 °C to remove insoluble material. Next, protein concentration was determined by a Bradford assay, and IP was performed from equal amounts of protein with α-HA monoclonal antibody (mAb) 12CA5 and Protein G Sepharose beads (GE Healthcare Life Sciences). Incubation was for 2 h at 4 °C while rotating. The precipitate was washed 3–5 times with NP-40 buffer, and precipitated proteins were eluted by boiling for 10 min in LDS NuPAGE sample buffer with DTT (Invitrogen). For MS analysis and analysis of p16 ubiquitination shown in Figs. 4D and 5A, N-ethylmaleimide was added to PBS used for washing the cell pellet, and to the NP-40 lysis buffer, to an end-concentration of 2 mM. After IP with the α-HA mAb 12CA5, the precipitate was eluted by boiling in denaturing SDS buffer. Next, SDS was quenched with NP-40 buffer, and α-HA IP was performed again, using anti-HA affinity matrix (3F10 mAb; Roche Applied Science), followed by elution in LDS sample buffer.

### Western blotting and antibodies

Protein was separated by SDS-PAGE using pre-cast 4–12% gradient NuPAGE gels (Invitrogen) according to the manufacturer’s protocol. Next, protein was transferred to nitrocellulose membrane by semi-dry blotting using the Trans-Blot Turbo system (Bio-Rad). Membrane was blocked in PBS with 5% w/v skim milk powder (Fluka, cat. No. 70166) or Roche blocking buffer (for α-FLAG probing) diluted in PBS, followed by antibody probing in 1% skim milk or Roche blocking buffer diluted in Tris-buffered saline (20 mM Tris–HCl pH 7.6, 150 mM NaCl) with 0.1% Tween-20 (TBST). Fluorescence signal was detected by the Odyssey Imaging System (LI-COR), chemiluminescence (Pierce Biotechnology) was detected by the ChemiDoc Imaging System (Bio-Rad) or by exposure to film (Kodak). Primary antibodies used were peroxidase-conjugated α-FLAG mAb M2 (Roche), DY-800 or DY-682 (Dyomics) fluorochrome-conjugated (conjugated in house) α-HA mAb 12CA5, mouse-α-NAT5 mAb M01, Clone 2C6 (NatB catalytic subunit; Abnova), rabbit α-P16 polyclonal antibody C-20 (pAb; Santa-Cruz sc-468) mouse-α-Tubulin (Sigma T6199) and rabbit α-GFP (pAb)^54^. Fluorochrome-conjugated goat-α-rabbit Ig (LI-COR) was used as secondary antibody.

### CHX-chase assay

Transfected HEK 293T cells were cultured in presence of 50 μg/ml CHX (Sigma), in some instances in combination with 50 μM MG132 (Calbiochem) for the indicated time periods. For immunoblot read-out, cells were harvested and lysed in NP-40 buffer, followed by immunoblotting. Read-out was by the Odyssey Imaging System (LI-COR) to allow for quantification. Signal intensity of HA-tagged substrate was normalized to signal intensity of co-expressed GFP. For flow cytometric read-out of GFP, cells were harvested by trypsinization, and GFP and BFP signal intensities were determined by analyzing cells using a plate reader on the LSR Fortessa (BD Biosciences). For both read-outs, signal at the 0 h time point was set to 100%. Mean and SEM of three independent experiments was plotted, and points were connected by a one-phase decay curve fit. Initial curve fitting was done to determine the lowest plateau. Subsequently, this plateau was set as constraint for all samples in the experiment, in addition to a Y0 = 100 constraint. Curve fitting and statistical analysis were performed using GraphPad Prism version 9.5.0 (Graph Pad software).

### Mass spectrometry

Proteins were immunoprecipitated as described above, followed by SDS-PAGE and staining of proteins in the gel using SimplyBlue SafeStain (Life Technologies), after which bands of interest were excised. To assess Bcl-B ubiquitination, N-methyl iodoacetamide (Sigma-Aldrich) was used as alkylation reagent instead of standard iodoacetamide, to avoid false-positive interpretation of ubiquitination^55^. Next, in-gel trypsin-digestion was performed using the Proteineer DP digestion robot (Bruker). Peptides were extracted from the gel, lyophilized, dissolved in 95/3/0.1 v/v/v water/acetonitril/formic acid and subsequently analyzed by on‐line nanoHPLC MS/MS using an 1100 HPLC system (Agilent Technologies), as previously described by Meiring et al.^56^. Peptides were trapped at 10 μl/min on a 15‐mm column (100‐μm ID; ReproSil‐Pur C18‐AQ, 3 μm, Dr. Maisch GmbH) and eluted to a 200 mm column (50‐μm ID; ReproSil‐Pur C18‐AQ, 3 μm) at 150 nl/min. All columns were packed in house. The column was developed with a 30‐min gradient from 0 to 50% acetonitrile in 0.1% formic acid. The end of the nanoLC column was drawn to a tip (5-μm ID), from which the eluent was sprayed into a 7‐tesla LTQ‐FT Ultra mass spectrometer (Thermo Electron). The mass spectrometer was operated in data‐dependent mode, automatically switching between MS and MS/MS acquisition. Full scan MS spectra were acquired in the FT‐ICR with a resolution of 25,000 at a target value of 3,000,000. The two most intense ions were then isolated for accurate mass measurements by a selected ion-monitoring scan in FT‐ICR with a resolution of 50,000 at a target accumulation value of 50,000. Selected ions were fragmented in the linear ion trap using collision‐induced dissociation at a target value of 10,000. In a post-analysis process, raw data were first converted to peak lists using Bioworks Browser software v3.2 (Thermo Electron), and then submitted to the Swissprot database, using Mascot v. 2.2.04 (www.matrixscience.com) for protein identification. Mascot searches were with 2 ppm and 0.5 Da deviation for precursor and fragment mass, respectively, and trypsin as enzyme. Collision‐induced dissociation spectra were manually inspected. With the exception of a few very clear ME2 spectra, in most cases peptides of interest were synthesized to confirm spectrum assignment.

### Statistics

Statistical tests used are indicated in the figure legends. The X number of biological repeats are indicated in the figure legends as “n = X”.

## Supplementary Information

Supplementary Information.

## Data Availability

All datasets generated or analysed during this study are available from the corresponding author on reasonable request.
