# Supplementary material for: N-terminal acetylation can stabilize proteins independent of their ubiquitination
Source: Sci Rep. 2023 Apr 1;13:5333. doi: 10.1038/s41598-023-32380-3 (PMC10067848; doi:10.1038/s41598-023-32380-3)
Supplement: Supplementary file 1 — Supplementary Information. [file 41598_2023_32380_MOESM1_ESM.pdf]

N-terminal acetylation can stabilize proteins independent of their ubiquitination

Bert van de Kooij, Evert de Vries, Rogier W. Rooswinkel, George M.C. Janssen, Frédérique K. Kok, Peter A. van Veelen and Jannie Borst

Supplementary information file

Supplementary Figure 1

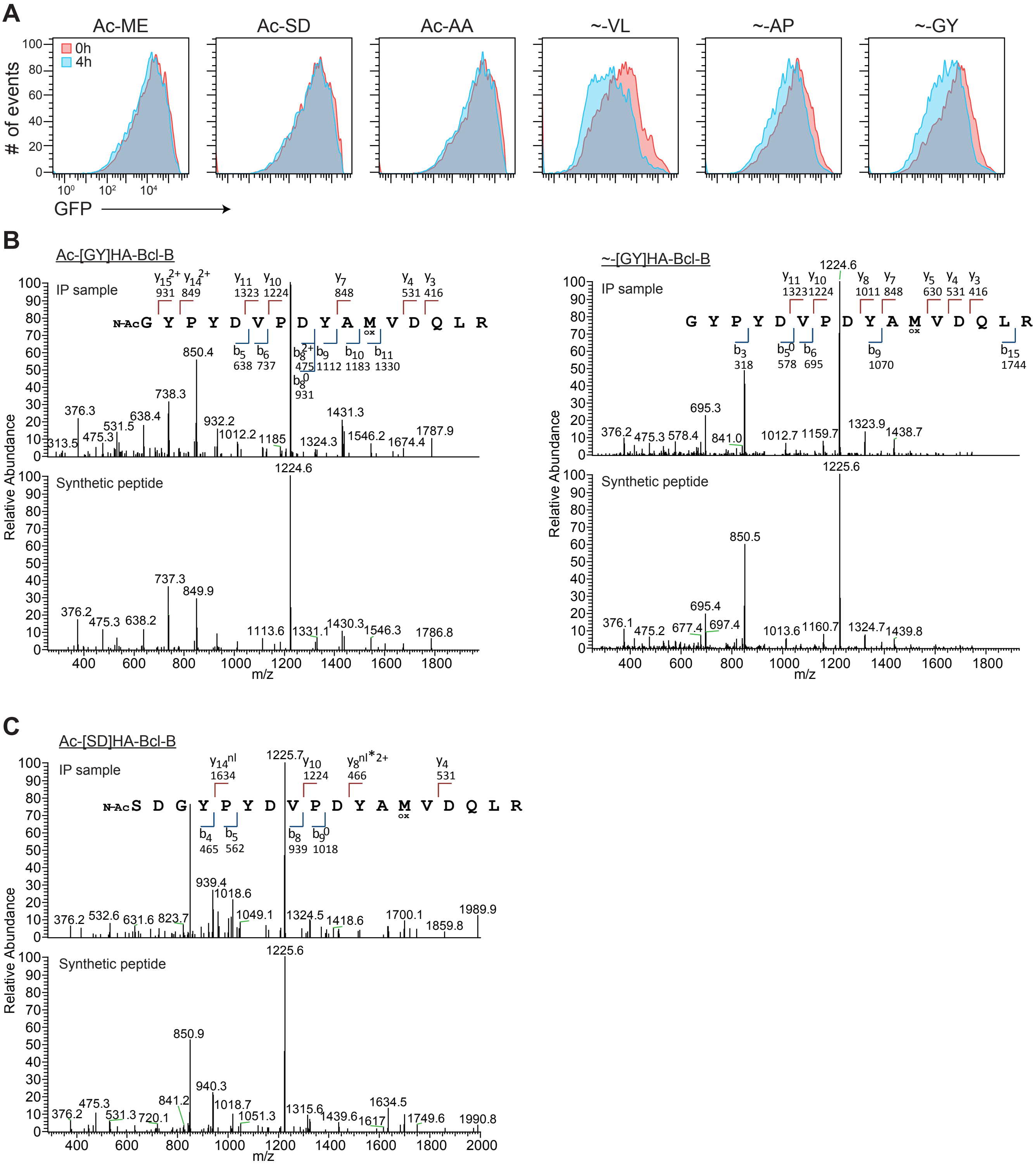

Supplementary Figure 1. Validation of the Nt-acetylation status of HA-Bcl-B. (A) HEK 293T cells were transfected to express BFP as a reference protein, together with HA-GFP variants carrying indicated N-terminal dipeptide sequences (”Ac-” = Nt-acetylated, “~” = Nt-acetyl-free). Cells were cultured in presence of CHX (50 μg/ml) for the indicated time periods, followed by flow cytometric analysis. Histograms show GFP intensity of all BFP positive cells, at the 0 h and 4 h CHX chase time point. **(B, C)** HEK 293T cells were transfected to express HA-Bcl-B variants carrying either a [GY] (panel B) or [SD] (panel C) N-terminal dipeptide sequence. The expressed proteins were isolated by a tandem HA-IP protocol and the precipitate was separated by SDS-PAGE. Bands of interest were excised followed by trypsin-digestion and analysis by LC-MS/MS. Depicted are the tandem mass spectra of the detected N-terminal tryptic peptides in the immunoprecipitate (IP sample; upper panel). To validate these spectra, a peptide-mimic was synthesized and analyzed (lower panel; 2+ = doubly charged, 0 = - H_2O, *=-NH_3, nl = neutral loss oxidized methionine).

Supplementary Figure 2

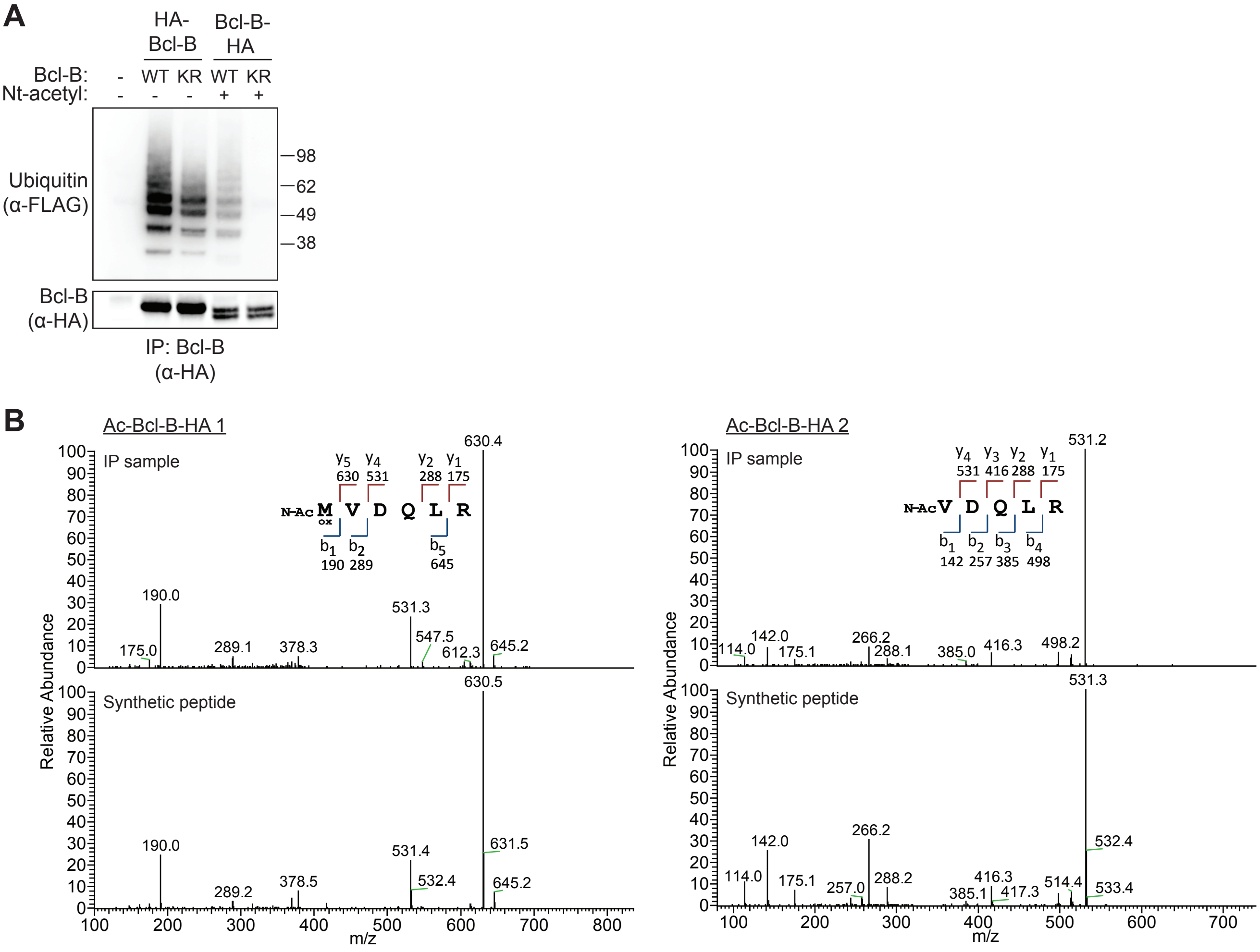

Supplementary Figure 2. The native N-terminus of Bcl-B is Nt-acetylated and not targeted for ubiquitination. (A) HEK 293T cells were transfected to express FLAG-tagged ubiquitin together with either wild-type (WT) Bcl-B, or a mutant in which all lysines were mutated to arginine (KR). Both Bcl-B variants were HA-tagged either N-terminally (HA-Bcl-B) or C-terminally (Bcl-B-HA). The transfected cells were lysed under denaturing conditions, followed by immunoprecipitation of Bcl-B and immunoblot analysis of the precipitates. **(B)** MS analysis of C-terminally HA-tagged Bcl-B, as outlined for Supplementary Figure 1B. Depicted are spectra of N-terminal peptides with (Ac-Bcl-B-HA 1) and without (Ac-Bcl-B-HA 2) initiator methionine.

Supplementary Figure 3

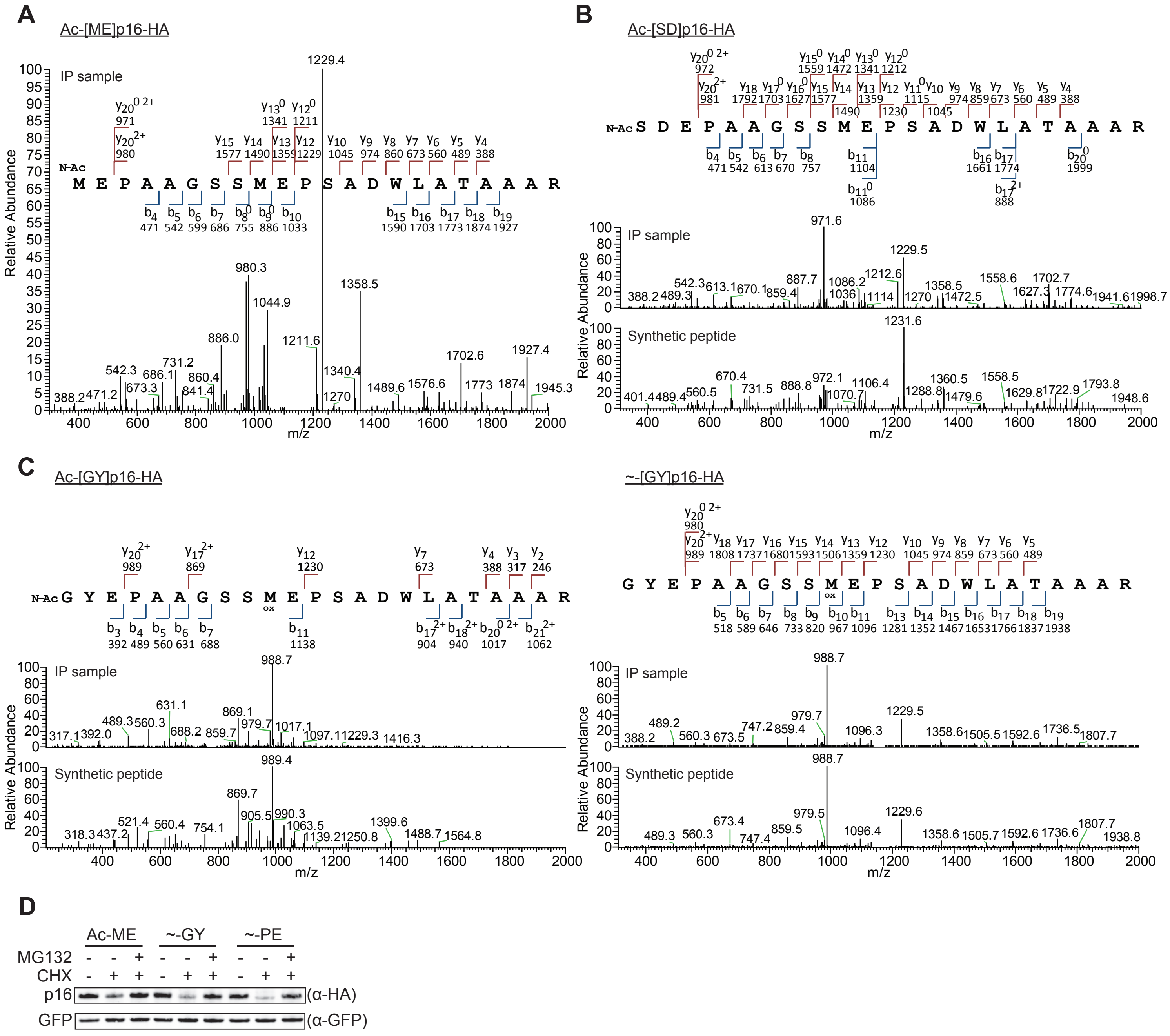

Supplementary Figure 3. Validation of the Nt-acetylation status of p16-HA variants. (A-C) MS analysis of p16-HA variants (2+ = doubly charged, 0 = - H_2O), as outlined in Supplementary Fig. 1B. **(D)** HEK 293T cells were transfected to express GFP as a stable protein control, together with p16-HA variants carrying the indicated N-terminal dipeptide sequences ("Ac-" = Nt-acetylated, "~" = Nt-acetyl-free). Next, the cells were left untreated, or treated for six hours with either CHX (50 μg/ml) alone, or CHX and MG132 (50 μM) combined. Cell lysates were analyzed by immunoblotting. A representative Western blot image of two independent experiments is shown.

Supplementary Figure 4

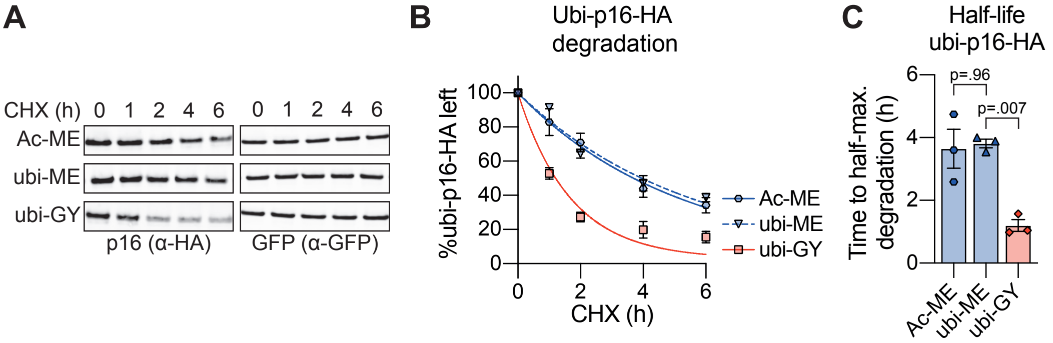

Supplementary Figure 4. Ubiquitin-fused p16 is stabilized by Nt-acetylation. (A, B) HEK 293T cells were transfected to express GFP as a reference protein, together with [ME]p16-HA or ubiquitin-HA-p16 fusion variants carrying either an [ME] or [GY] dipeptide sequence at the N-terminus of p16 ("Ac-"- = Nt-acetylated, "ubi" = ubiquitin fusion). Cells were cultured in presence of CHX (50 μg/ml) for the indicated time periods, followed by immunoblot analysis. P16-HA signals were quantified and corrected for GFP signal intensity, and the resulting value at the 0 h time point was set to 100%. Data points were connected by a one-phase decay curve fit. Panel A shows a representative Western blot image, panel B shows the quantification (n=3; mean ± SEM). **(C)** Based on the data in panel B, the half-lives of the indicated p16-HA variants were determined (n=3; mean ± SEM, One-way ANOVA, post hoc Dunnett's).

Supplementary Figure 5

A

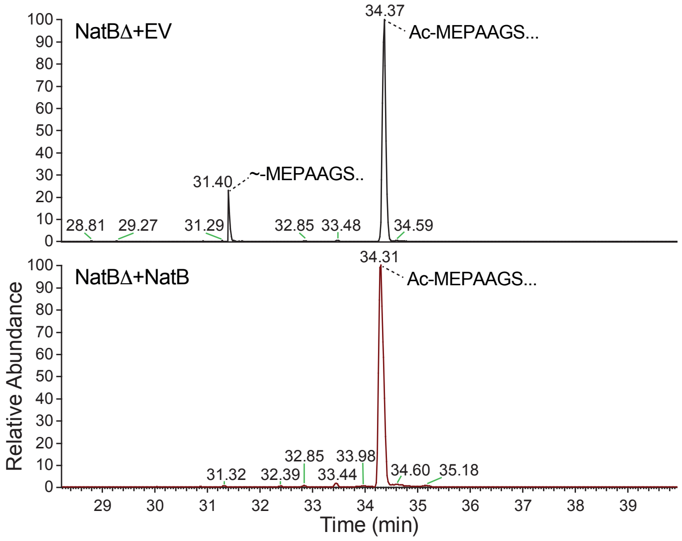

B

Cell-line	Confidence	Annotated sequence	Modification	Relative abundance (%)
NatBΔ +EV	High	[-].MEPAAGSSMEPSADWLATAAAR.[G]	1xAcetyl [N-Term]; 2xOxidation	72
NatBΔ +EV	High	[-].MEPAAGSSMEPSADWLATAAAR.[G]	2xOxidation	28
NatBΔ +NatB	High	[-].MEPAAGSSMEPSADWLATAAAR.[G]	1xAcetyl [N-Term]; 2xOxidation	88
NatBΔ +NatB	High	[-].MEPAAGSSMEPSADWLATAAAR.[G]	1xAcetyl [N-Term]; 1xOxidation	12

C

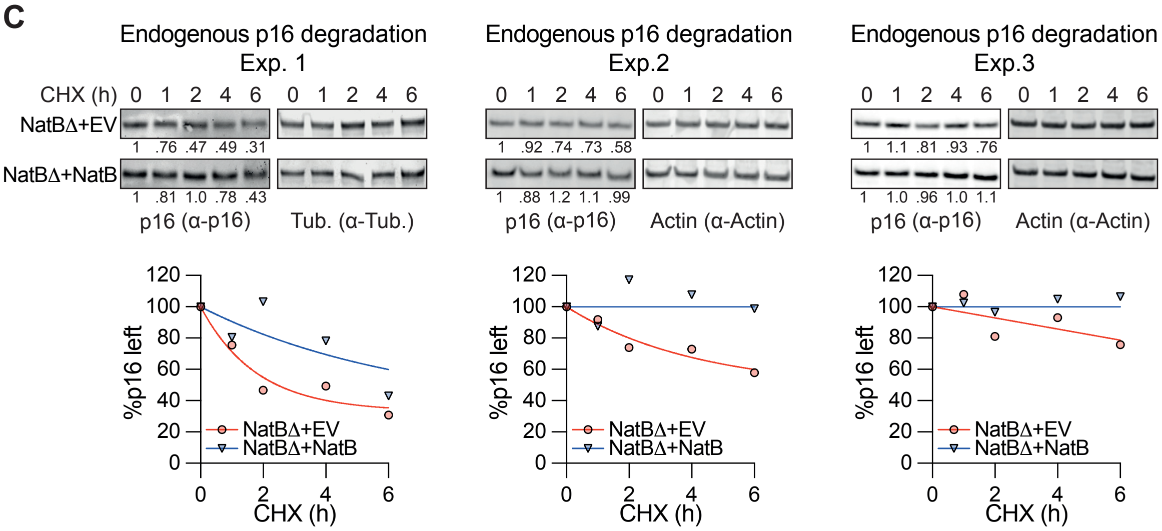

Supplementary Figure 5. Loss of NatB increases the degradation rate of endogenous p16. (A, B) [ME]p16-HA was expressed in the HEK 293T NatBΔ cell-lines described in figure 6. Next, the Nt-acetylation status was determined by LC-MS/MS. Panel A shows the summed mass chromatograms of the Nt-acetyl-free (m/z 751.337) and Nt-acetylated (m/z 765.341) N-terminal peptide (MEPAAGSSMEPSADWLATAAAR), showing the extent of acetylation in the NatBΔ +EV (upper trace) and NatBΔ +NatB condition (lower trace). Panel B shows the quantification of the relative abundance of each modified N-terminal peptide as identified by MS/MS. **(C)** Western blots (upper panel) and quantification (lower panel) of each of the three independent experiments that were averaged and plotted in figure 6D. Values below the α-p16 western blots indicate the signal intensity for each p16 band, corrected for input and normalized to the 0h time point.

Supplementary Figure 6

Figure 2C

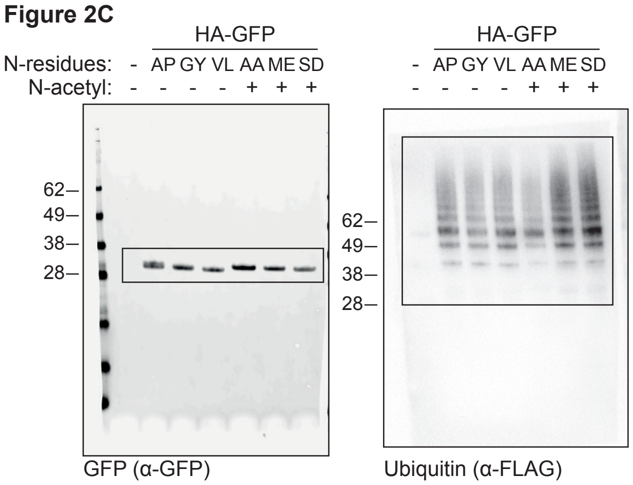

Figure 3A

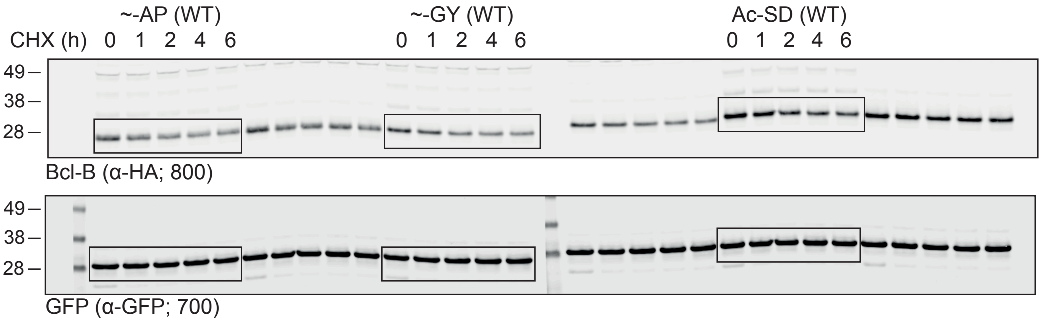

Figure 3C

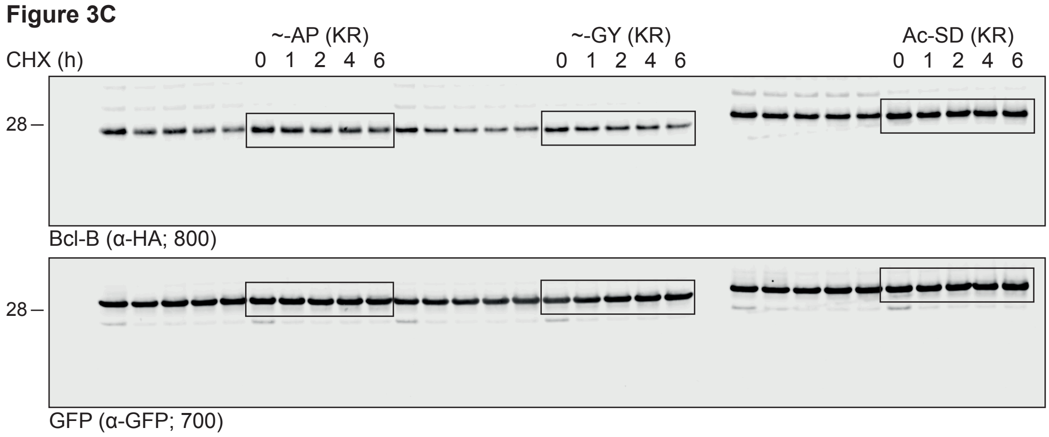

Supplementary Figure 6. Original, uncropped Western blot images. Depicted are the original western blot images, uncropped, for the indicated figure panels. Boxed areas indicate cropping outline. Note that for figures 3A, 3C, 4A and 5B, as well as for supplementary figures 3D and 4A, only part of the membrane was scanned on the Odyssey imaging system. In these cases, the original, uncropped image containing the complete scanned area is shown. In case of figures 3C, 4A and 5B the marker was absent, and molecular weight indication is an estimation based on similar blots. For figure 3F, an additional dark exposure is included to more clearly show the edges of the western blot membrane. For figure 4F the original western blot image could not be retrieved.

Supplementary Figure 6, continued

Figure 3F

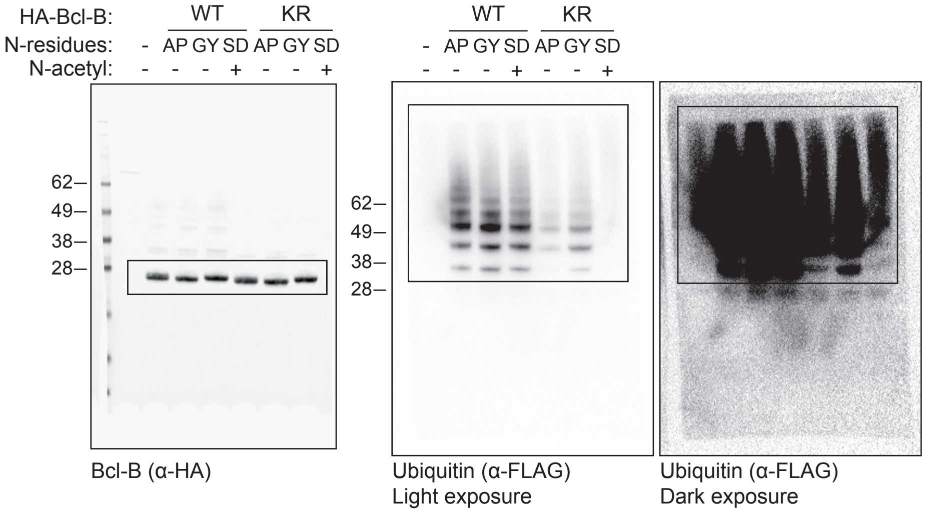

Figure 4A and Figure 5B

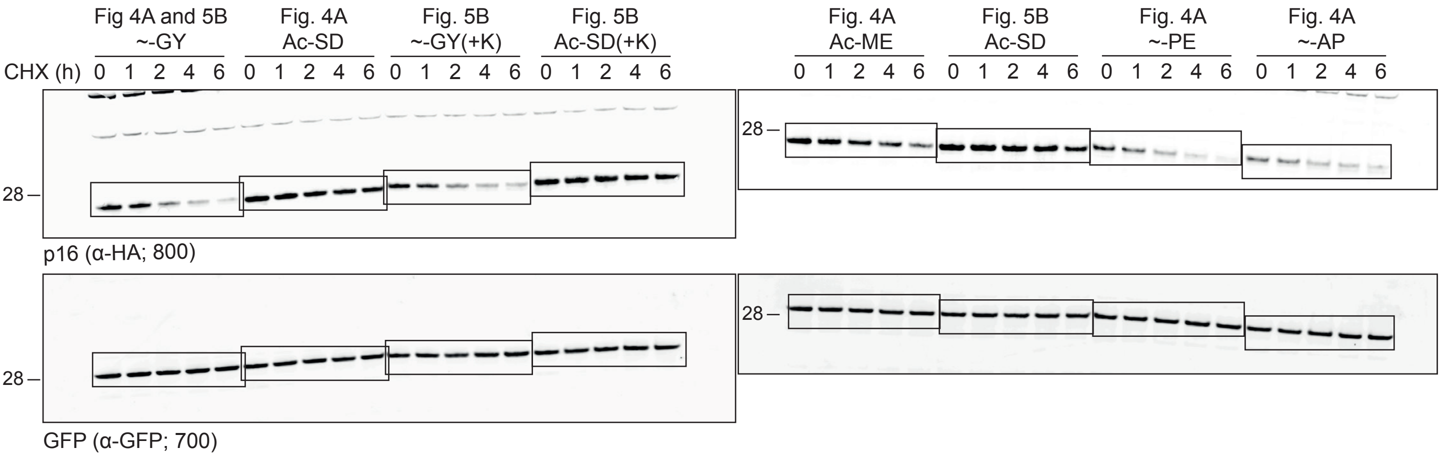

Figure 4D part 1

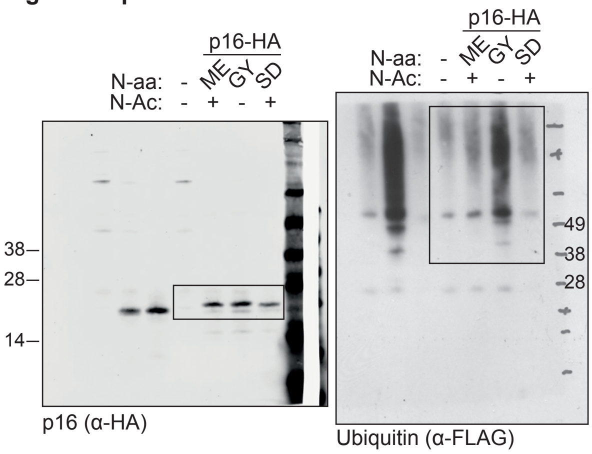

Figure 4D part 2 and Figure 5A

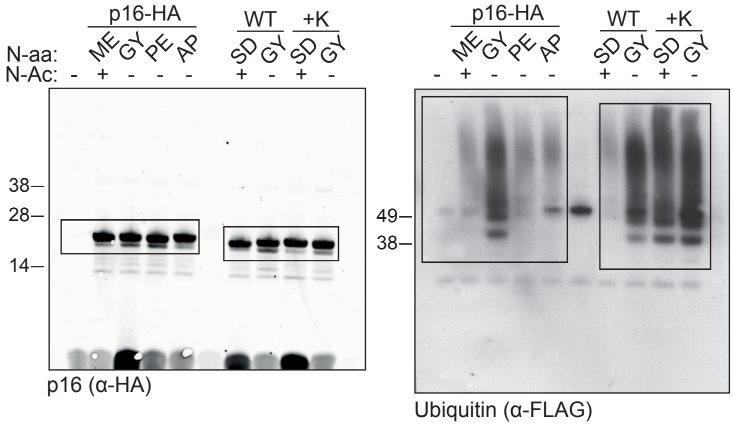

Figure 6B

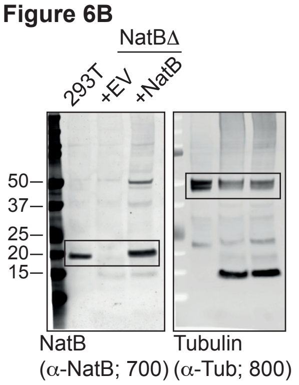

Supplementary Figure 6, continued. Original, uncropped Western blot images.

Supplementary Figure 6, continued 2

Figure 6C

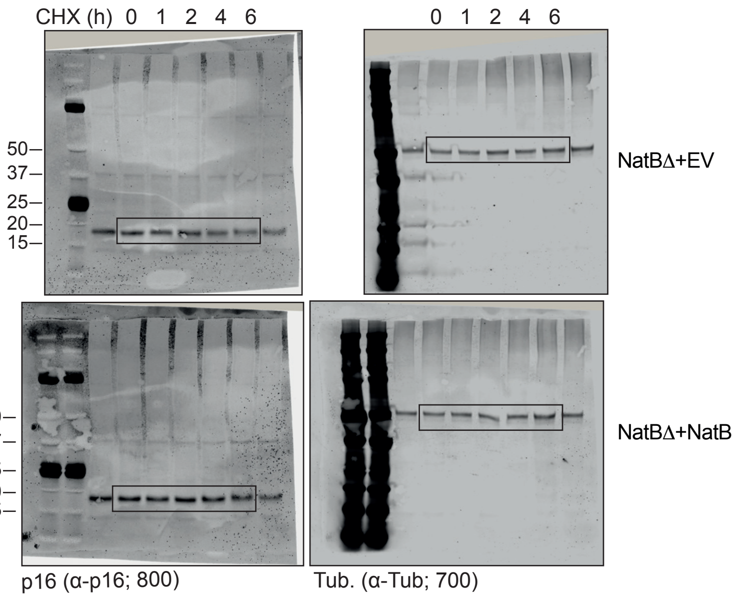

Supplementary Figure 2A

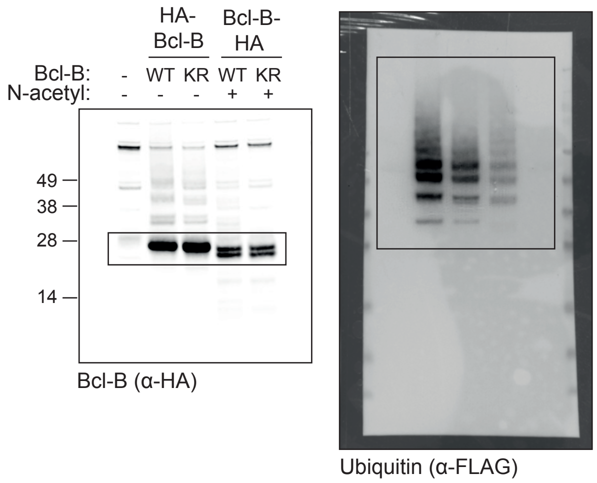

Supplementary Figure 3D

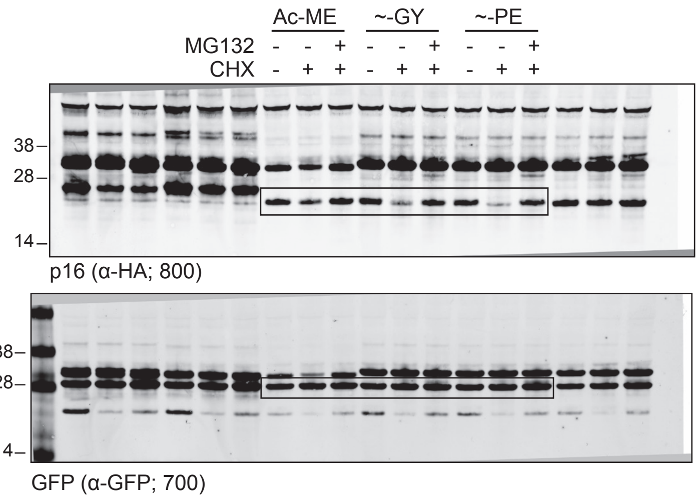

Supplementary Figure 4A

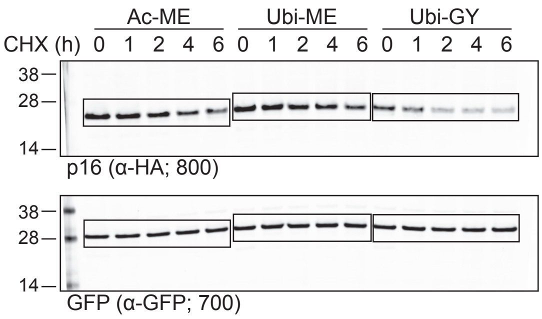

Supplementary Figure 5C_exp. 2

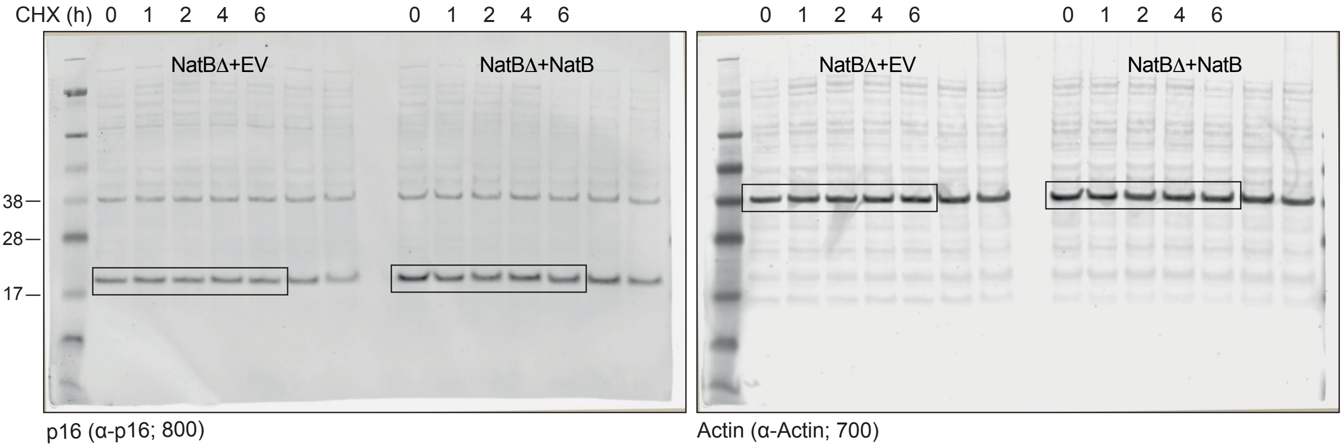

Supplementary Figure 6, continued 2. Original, uncropped Western blot images.

Supplementary Figure 6, continued 3

Supplementary Figure 5C_exp. 3

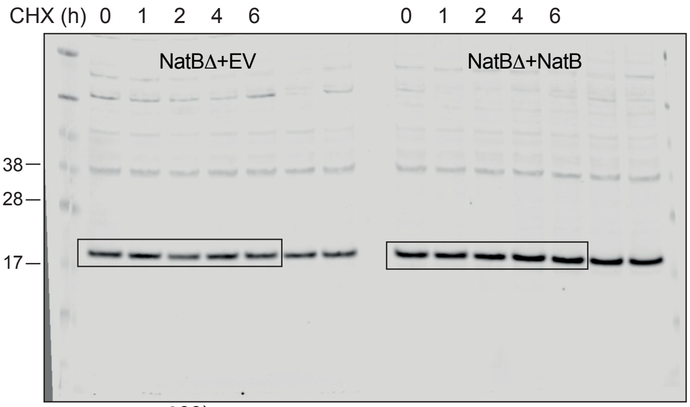

p16 (α-p16; 800)

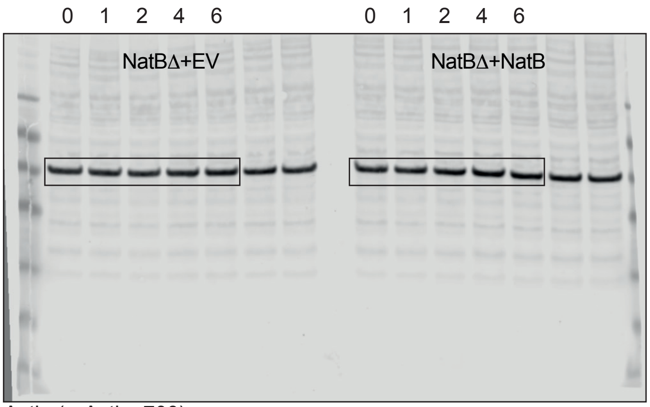

Actin (α-Actin; 700)

Supplementary Figure 6, continued 3. Original, uncropped Western blot images.
